# Recent advances in fluorescent probes for guided surgery of head and neck carcinomas

**DOI:** 10.1039/d6ra04409c

**Published:** 2026-07-10

**Authors:** Pavol Karabínoš, Robert Pola, Tomáš Etrych

**Affiliations:** a Institute of Macromolecular Chemistry AS CR Heyrovského nám. 1888/2 162 00 Prague 6 Czech Republic karabinos@imc.cas.cz pola@imc.cas.cz etrych@imc.cas.cz

## Abstract

Head and neck squamous cell carcinoma (HNSCC) rank as the sixth most common cancer worldwide. Traditional treatments include surgery, radiation, and chemotherapy, with surgery being the primary option for early-stage disease. However, achieving clear surgical margins is a major challenge, as incomplete removal of tumor cells increases the risk of recurrence and metastasis, negatively affecting patient survival. This often requires extensive tissue removal, leading to complications such as swallowing and speech difficulties. However, it is difficult for surgeons to accurately distinguish the tumor tissue and its boundary with the naked eye. Consequently, fluorescence-guided surgery (FGS) has attracted increasing attention in recent years. FGS presents a promising solution by providing high sensitivity, real-time visualization of tumor margins, allowing for more precise and less invasive procedures, thereby preserving patient function and improving quality of life. This review presents a structured overview of recent fluorescent probe development for HNSCC surgery, with additional context on foundational advances and recent clinical applications. Across the reviewed studies, EGFR-targeted probes achieved reported tumor-to-background ratios (TBRs) of up to 7.1, PARP-targeted probes up to 10.3, FAP-targeted probes approximately 5.9, GLUT1-targeted probes approximately 2.1, and Hsp70-targeted probes approximately 2.6. Among these approaches, EGFR-targeted probes currently represent the most clinically advanced class, with agents such as panitumumab-IRDye800CW and cetuximab-IRDye800CW already evaluated in clinical studies. In contrast, activatable probes and dual-modality imaging agents represent emerging strategies with the potential to further improve tumor specificity and intraoperative contrast.

## Abstract 2

Recent advances in fluorescent probes for fluorescence-guided surgery, featuring diverse probe designs, targeting approaches, and imaging strategies.

## Graphical abstract

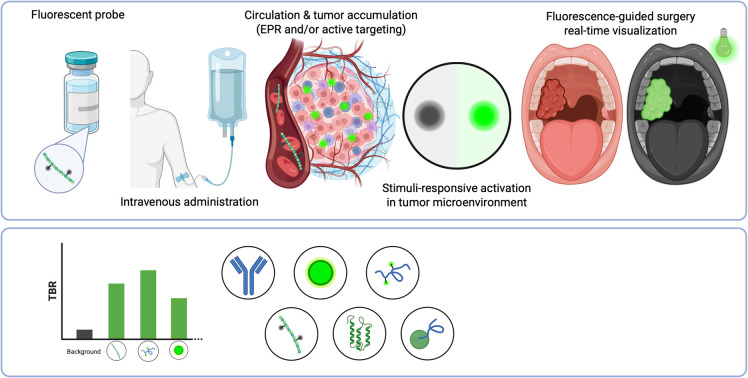

## 1. Introduction

Head and neck squamous cell carcinoma (HNSCC) is the sixth most common type of cancer globally, with over 940 000 new cases annually. HNSCC accounts for roughly 4.7% of all cancer diagnoses and deaths.^1,2^ Traditional treatment modalities for these cancers typically include surgery, radiation therapy, and chemotherapy, either as monotherapies or in combination.^3–9^ Among these, surgical resection remains the cornerstone for curative intent, especially in the early stages of the disease. However, achieving clear surgical margins is a critical challenge in head and neck cancer surgeries. Incomplete resection or residual tumor cells at the surgical margins significantly increase the risk of local recurrence and metastasis, adversely impacting patient survival. Therefore, the radicality of the procedure is a key factor for surgical resection of HNSCC, necessitating the removal of sufficient cancer tissue, even at the cost of removing nearby healthy tissue.

**Fig. 1:**
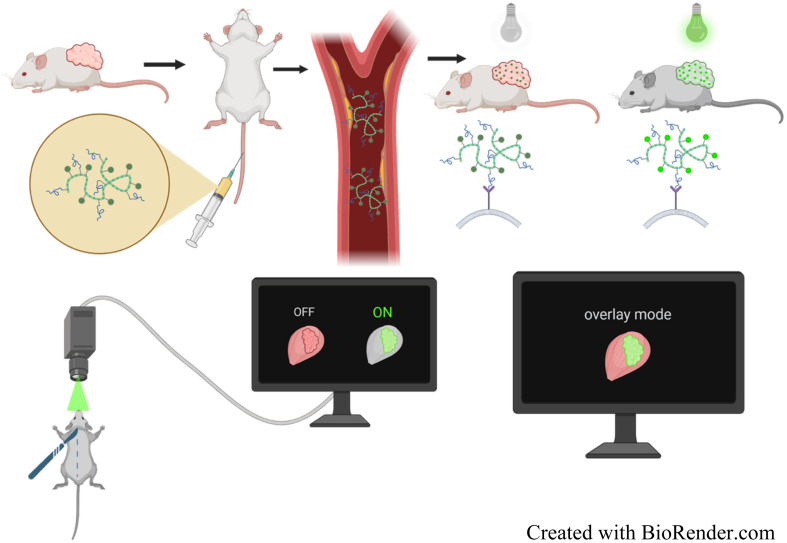
Schematic illustration of the principle of intraoperative tumor imaging using fluorescent probes. After intravenous administration, the fluorescent probe circulates in the bloodstream and accumulates within the tumor by binding to its specific molecular target. Upon illumination with excitation light, the probe emits a fluorescent signal that highlights the tumor tissue. This enables real-time intraoperative visualization of malignant tissue. The surgeon can observe the fluorescence either in standard fluorescence mode (ON/OFF) or in overlay mode, where the fluorescent signal is projected onto the normal white-light image to guide precise tumor delineation and resection.

Unfortunately, this approach carries the risk of various negative effects, such as swallowing problems, speech and articulation disorders. Currently, surgeons rely largely on visual inspection with the naked eye, aided by intraoperative frozen-section analysis, to guide tumor resection.^10–14^ These limitations underscore the need to improve surgical outcomes and reduce the morbidity and mortality associated with these malignancies. This need has led to increased attention in the field of FGS. FGS offers a novel approach to address this challenge by providing real-time visualization of tumor margins during the surgical procedure [Fig. 1]. These probes can selectively bind to tumor cells and emit fluorescence under specific light wavelengths, thereby delineating cancerous tissues from healthy ones with high precision. The integration of fluorescent probes into surgical practice not only enhances the surgeon's ability to achieve meticulous removal of malignant tissues but also minimizes the need for extensive resections, thereby preserving function and quality of life for patients.^15–17^ In summary, an ideal fluorescent probe for intraoperative FGS of head and neck carcinomas should have properties of high specificity, sensitivity, an excellent tumor-to-background ratio, and effective tumor accumulation without toxic effects on the patient, underscoring the current challenge of designing such probes with strong biocompatibility.^15,17^ Fluorescent dyes incorporated into targeted probes used in FGS significantly enhance tumor visualization for surgeons. These dyes provide deep tissue penetration by emitting fluorescence in the near-infrared (NIR-I or NIR-II) spectrum. Their suitability is further supported by low toxicity, high biocompatibility, and compatibility with imaging equipment.^18–24^ The summarizes of the fluorescent dyes and probes discussed in this review is depicted in [Table 1].^25–37^

**Table 1:** Table of fluorescent dyes used in fluorescence-guided surgery for head and neck carcinomas

Dye	Emission peak	Absorption peak
Indocyanine green	820 nm	780 nm
IRDye 800CW	796 nm	775 nm
Cyanine 7	775 nm	750 nm
IR-820	820 nm	690 nm
SQ890	1000–1700 nm	890 nm
BODIPY-FL	520 nm	470 nm
Coumarin derivative	520 nm	462 nm
SOD9-TPP	660 nm	535 nm
Dy 633	658 nm	637 nm

The objective of this review is to provide a comprehensive overview of recent advances in fluorescent probes for FGS of HNSCC. While the primary focus is on novel probe development, these advances are discussed within the context of foundational technologies and clinically investigated imaging agents. Although several reviews have recently addressed FGS in head and neck cancer,^38–40^ they have primarily focused on general imaging principles or clinically investigated tracers. In contrast, substantial progress has been made in the development of fluorescent probes, activatable imaging agents, and tumor-targeted strategies. This review provides an updated overview of these developments, with particular emphasis on their application in FGS of HNSCC. Given the rapid expansion of research in this area and the increasing diversity of probe designs, a comprehensive synthesis of available evidence is needed to facilitate comparison between emerging approaches, identify current challenges, and guide future translational and clinical research. The present review aims to: (1) summarize recent advances in fluorescent probes and emerging imaging strategies for FGS of HNSCC; (2) compare the different targeting approaches, probe designs, and activation mechanisms employed for tumor visualization; and (3) discuss their advantages, limitations, and potential for future clinical translation.

## 2. Foundations of fluorescence in head and neck surgery

The clinical interest in FGS for HNSCC has grown steadily over the past two decades. This is largely driven by the anatomical complexity of the head and neck region and the high risk of incomplete tumor resection, a well-documented predictor of local recurrence and decreased survival.^38,41^ Although FGS was initially explored in other surgical fields, its relevance to head and neck oncology became apparent due to the difficulty of visually or palpably distinguishing tumor margins from surrounding normal tissue during resection.^42,43^ Early efforts relied on autofluorescence and non-specific dyes such as fluorescein and indocyanine green (ICG). While ICG offered good tissue penetration and was adopted for sentinel lymph node mapping and perfusion monitoring, its lack of tumor specificity limited its usefulness in oncologic surgery.^44,45^ These shortcomings prompted the development of molecularly targeted fluorescent probes, which aim to selectively bind to markers overexpressed in malignant cells. One of the earliest and most widely studied targets is the epidermal growth factor receptor (EGFR), which is overexpressed in approximately 90% of HNSCCs and is associated with poor prognosis, treatment resistance, and increased recurrence rates.^38,46,47^ This led to the design of antibody-based imaging agents such as cetuximab-IRDye800CW and panitumumab-IRDye800CW, or polymer-based probes,^48–50^ which target EGFR and enable high-contrast tumor visualization during surgery. These developments laid the groundwork for the next generation of fluorescence probes, many of which are currently under development and aimed at improving surgical precision in head and neck oncology.

## 3. Current clinical applications of fluorescent probes in head and neck oncology

Fluorescence guided surgery is increasingly transitioning from experimental imaging to clinical application in the treatment of HNSCC. This progress is driven by a growing body of clinical studies evaluating molecularly targeted fluorescent probes capable of intraoperatively highlighting tumor tissue with high specificity. Among the most clinically advanced agents is panitumumab-IRDye800CW, which combines a fully human monoclonal antibody targeting the EGFR with a near-infrared fluorophore. Given the high prevalence of EGFR overexpression in HNSCC, this agent has been evaluated in Phase I clinical trials, demonstrating safety and enhanced tumor margin visualization in HNSCC, and has advanced into early Phase II evaluation.^43,51,52^ In one pivotal study, real-time imaging with panitumumab-IRDye800CW enabled precise delineation of tumor margins and even detection of microscopic residual disease not visible to the naked eye.^51^ Similar agents, such as cetuximab-IRDye800CW, have also demonstrated benefit in visualizing tumor borders, particularly in oral cavity and oropharyngeal resections. These conjugates offer reliable signal stability and deep tissue penetration within the NIR-I window, allowing for continuous intraoperative feedback without disrupting the surgical field.^38^ Additional clinically investigated targeted probes have also emerged in recent years. ABY-029, an anti-EGFR affibody conjugated to IRDye800CW, has demonstrated favorable safety and imaging characteristics in early clinical studies and represents a promising alternative to full-length antibody-based tracers due to its smaller size and more rapid pharmacokinetics.^53^ Furthermore, PARPi-FL, a fluorescent PARP1-targeted probe, has progressed from successful preclinical evaluation to Phase I clinical testing in patients with oral squamous cell carcinoma,^54^ demonstrating the feasibility of molecularly targeted fluorescence imaging beyond EGFR-based approaches. These developments highlight the growing diversity of fluorescent imaging agents currently undergoing clinical translation in HNSCC and related malignancies. In addition to targeted fluorescent probes, other fluorescence-based approaches are also being explored for HNSCC surgery. A recent prospective pilot clinical study investigated 5-aminolevulinic acid (5-ALA), an FDA-approved precursor of the heme biosynthesis pathway that is converted intracellularly to the fluorescent metabolite protoporphyrin IX. In 2024, Filip *et al.* administered oral 5-ALA to seven patients undergoing surgical resection of HNSCC and evaluated intraoperative fluorescence using 405 nm blue light excitation. Six of seven tumors demonstrated robust fluorescence, enabling visualization of primary tumors as well as positive surgical margins, perineural invasion, severe dysplasia, and metastatic lymph nodes. Rather than targeting a specific molecular receptor, 5-ALA exploits differences in tumor metabolism that result in preferential accumulation of fluorescent protoporphyrin IX within malignant tissue. These findings demonstrate the feasibility of 5-ALA-assisted fluorescence imaging as a complementary approach for intraoperative tumor visualization and margin assessment in HNSCC.^55^ Despite these advances, only a few probes have progressed to late-stage clinical trials, and none are yet approved for routine standard-of-care use.^38,43^ Non-targeted fluorophores, such as indocyanine green (ICG), remain in off-label use for perfusion imaging and sentinel lymph node mapping but lack tumor specificity.^18,56^ These limitations have reinforced the demand for biomarker-specific agents targeting proteins such as EGFR, PARP1, or GLUT1.^45,46^ Furthermore, fluorescence imaging is being incorporated into transoral robotic surgery (TORS), enabling enhanced visualization in anatomically complex regions like the oropharynx. Preliminary trials suggest that FGS may not only support margin clearance but also reduce unnecessary excision of healthy, functional tissue a consideration of particular relevance in the tongue base, tonsillar pillars, and supraglottic larynx.^43^

## 4. Methodology of literature selection and analysis

**Fig. 2:**
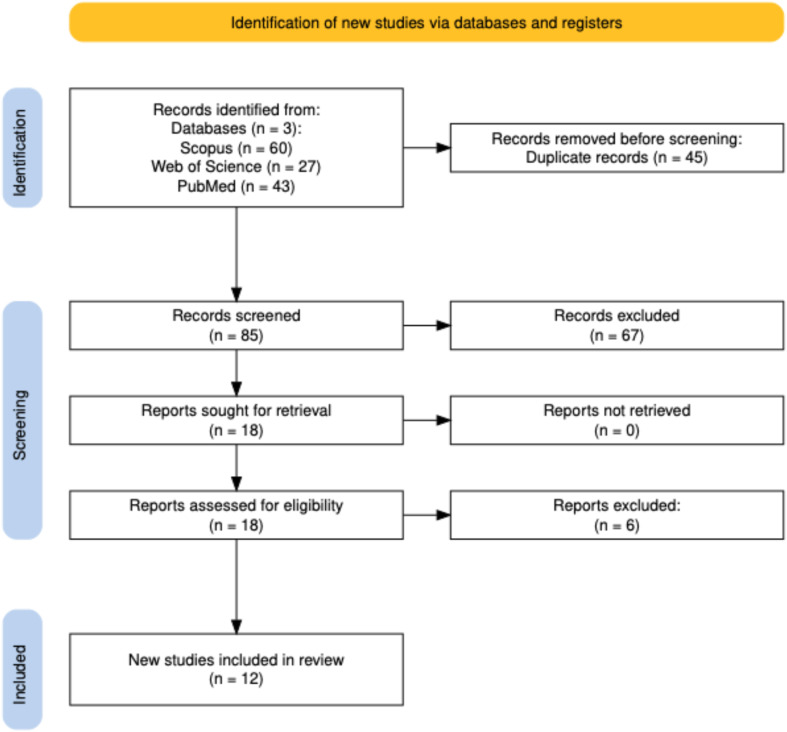
PRISMA flow diagram illustrating the study selection process for articles included in this review.

A targeted literature review was conducted to support the analysis of recent developments in fluorescent probe design for HNSCC surgery. Specifically, a comprehensive search was performed to identify recent articles discussing the use of fluorescent probes for FGS of HNSCC. The primary databases used for this search were Web of Science, PubMed, and Scopus. The search was restricted to articles published from 2020 to the present for the identification of fluorescent probes included in this review. No publication-year restriction was applied to studies cited for background information and contextual discussion. To ensure the relevance and quality of the articles reviewed, the following inclusion criteria were established: the articles must discuss the application of fluorescent probes in the context of HNSCC, and only studies that included *in vivo* distribution of the probes were considered. The most critical metric for our evaluation was the tumor-to-background ratio. The following search terms were used in various combinations to identify relevant articles: “fluorescent probes”, “guided surgery”, “head and neck”, and “HNSCC”. In each database, the search was tailored to meet the specific functionalities and search algorithms of the platform. The search identified 130 records across the three databases. After removal of duplicate records and screening according to the predefined eligibility criteria, 12 studies were included in the final analysis. The literature search and study selection process is summarized in [Fig. 2]. For each article that met the inclusion criteria, the following data were extracted: the type of fluorescent probe, probe design and composition, methodology of the *in vivo* distribution studies, reported tumor-to-background ratio, and key findings. The data were analyzed to compare the different types of fluorescent probes used in these studies, assess the methodologies employed for *in vivo* distribution studies and evaluate the reported tumor-to-background ratios to determine the effectiveness of each fluorescent probe. This approach allowed us to compare the current state of fluorescent probes use in HNSCC, providing valuable insights into the most promising probes and methodologies for future research and clinical application.

## 5. Types of fluorescent probes used for head and neck carcinomas

### 5.1. Always-on probes *vs.* activatable

Fluorescent probes are essential tools in biological and medical research, used to detect and visualize specific molecules within cells and tissues. These probes emit fluorescence upon excitation by light, enabling the study of biological processes in real-time with high sensitivity and specificity. Fluorescent probes can be broadly classified into two categories: always-on probes and activatable probes [Fig. 2]. Always-on probes are constantly fluorescent regardless of the presence of the target molecule or environmental conditions. These probes emit a steady fluorescent signal upon excitation, providing continuous visualization.

Activatable probes, in contrast, are designed to emit fluorescence in the presence of a specific target or under specific conditions. They are initially non-fluorescent or weakly fluorescent, and their fluorescence is “activated” by interactions with the target molecule, such as binding to a specific biomolecule,^57^ undergoing a chemical reaction,^50^ experiencing a change in the local environment (*e.g.*, pH,^50^ or intracellular levels of molecules like glutathione^57^). This selective activation minimizes background fluorescence and enhances the signal-to-noise ratio, making activatable probes highly useful for detecting specific molecular patterns with high precision. To increase the effectiveness of probes, active targeting might be used. There are certain molecular targets that can be leveraged to actively guide fluorescent probes toward tumor tissue, thereby improving their specificity. By conjugating fluorophores to ligands, such as peptides or antibodies, that selectively bind to overexpressed, tumor-associated markers, these molecularly targeted probes preferentially accumulate at malignant sites, ultimately enhancing tumor contrast and aiding in more precise surgical delineation. Several such molecular targets have been identified, and the specific probes that utilize them will be discussed in more detail below.^29,30,48,50,58–62^

### 5.2. Always-on probes

Firstly, we will consider some always-on probes investigated in this context, categorized according to their targeting strategies.

#### 5.2.1. Epidermal growth factor receptor

The EGFR is a transmembrane protein belonging to the receptor tyrosine kinase family. It regulates various cellular mechanisms, including cell growth, differentiation, and proliferation. EGFR is often overexpressed in several human carcinomas, including HNSCC. Due to its overexpression in these cancers, EGFR is considered an ideal target for tumor imaging purposes.^63–68^

In 2020, Pal *et al.* prepared a fluorescent probe, panitumumab-IRDye800CW, by conjugating the FDA-approved therapeutic antibody panitumumab which targets the EGFR with the near-infrared dye IRDye800CW.^58^ After conjugation the probe retains panitumumab's inherent ability to bind specifically to EGFR on the surface of cancer cells, while the IRDye800CW provides fluorescence detectable using imaging systems. This dual-function design enables the visualization of tumor tissues during surgical procedures. Instead of relying solely on total fluorescence intensity, Pal *et al.* focused on fluorescence lifetime (FLT). They found that the FLT within EGFR-overexpressing cancer cells was significantly longer than that of normal tissues, providing a high degree of sensitivity and specificity. By exploiting these photophysical properties, rather than just raw fluorescence intensity, the researchers were able to greatly enhance tumor contrast. This approach effectively reduces the confounding effects of nonspecific fluorescence uptake, which can occur due to poor targeting specificity. Preclinical evaluations began with *in vitro* assays using EGFR-overexpressing head and neck cancer cells. These cells exhibited significantly longer FLTs (approximately 0.75 ns) when treated with panitumumab-IRDye800CW compared to controls with non-specific antibodies or buffer, confirming the probe's specificity. In *in vivo* experiments involving FaDu tumor xenografts in mice, FLT-based tumor-to-normal tissue classification achieved 99% accuracy, far exceeding the 71% accuracy observed with fluorescence intensity alone. These results underscored the value of FLT in reducing the effects of heterogeneous nonspecific uptake. Subsequent clinical studies focused on oral squamous cell carcinoma (OSCC). Patients received systemic injections of panitumumab-IRDye800CW 48 hours before surgery. Postoperative tissue specimens underwent fluorescence lifetime imaging microscopy (FLIM), revealing consistently longer FLTs in EGFR-overexpressing tumor regions than in adjacent normal tissues. Immunohistochemistry validated that these longer FLTs spatially correlated with high EGFR expression. Sensitivity and specificity exceeded 98%, highlighting the method's strong diagnostic performance. Furthermore, FLT provided a standardized measure that did not fluctuate with imaging-system settings or differences in tissue composition, thereby offering a reliable tool for real-time surgical guidance.

In 2020, Pola *et al.* designed, synthesized, and systematically evaluated a series of fluorescent polymer probes to target EGFR in HNSCC cells (FaDu).^48^ These probes were built on (*N*-(2-hydroxypropyl)methacrylamide)-based copolymers (pHPMA), which are known for their biocompatibility and ability to accumulate selectively in tumors due to the enhanced permeability and retention (EPR) effect. Copolymers were labelled with the near-infrared dye Cyanine7 (Cy7) and conjugated with different EGFR-binding agents: the oligopeptide GE-11 (a known EGFR ligand^69,70^), human EGF (the natural ligand^71^), or the monoclonal antibody cetuximab (an FDA-approved EGFR inhibitor). The team also prepared high molecular weight polymer probes without any targeting ligands as controls. To achieve uniform polymer size distributions crucial for consistent biodistribution they employed free radical and Reversible Addition–Fragmentation chain Transfer (RAFT) polymerization. Authors examined the relationship between the polymer molecular weight and its tumor accumulation, comparing low (26 kDa), intermediate (170 kDa), and high (700 kDa) molecular weight probes at various times post-injection (15 minutes, 4 hours, and 24 hours). While the 26 kDa probe showed poor tumor fluorescence and was largely cleared by the kidneys, the 170 kDa and 700 kDa variants accumulated more effectively in tumors, particularly at the 4 and 24 hours time points. This observation underscored the importance of avoiding molecular weights below the renal filtration threshold (∼50 kDa (ref. 72)) when designing tumor-targeting nanoprobes. Building on these results, the authors proposed that polymer probes around 200 kDa offer the best balance between enhanced permeability and retention (EPR)-mediated tumor accumulation and sufficient clearance. In addition to passive targeting, Pola *et al.* assessed active EGFR binding. Although *in vitro* assays showed that cetuximab-conjugated probes possessed the highest EGFR-binding affinity, the *in vivo* data indicated that GE-11-based nanoprobes reached particularly high tumor accumulation by 24 hours. In contrast, hEGF-conjugated probes performed similarly to the non-targeted controls. The relatively lower *in vitro* affinity of GE-11 did not hinder its overall *in vivo* efficacy, likely because of prolonged circulation times that enabled sufficient probe internalization by tumor cells. The kinetics of tumor uptake also varied among targeting strategies. Cetuximab-conjugated probes rapidly localized to tumors—visible as early as 15 minutes post-injection—making them advantageous if quick surgical intervention is required. However, by 24 hours, GE-11-functionalized probes slightly surpassed cetuximab in overall tumor accumulation, suggesting that GE-11 is well suited for scenarios in which there is a longer interval between probe administration and surgery. Given that GE-11-based probes are simpler and potentially more cost-effective to synthesize, the study highlights how different clinical timelines could favour one targeting strategy over the other.

**Fig. 3:**
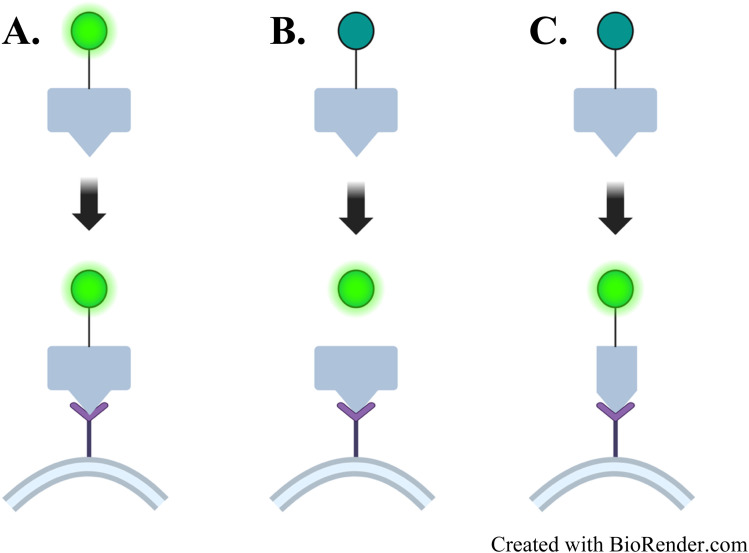
Schematic illustration of different types of fluorescent probes for tumor imaging. (A) Always-on probes emit fluorescence continuously and highlight the tumor upon accumulation at the target site. (B) Activatable probes remain quenched until they undergo specific enzymatic or microenvironmental activation, releasing the fluorophore and switching on the fluorescence. (C) Activatable probes undergo a structural or chemical transformation upon binding or in response to the tumor microenvironment, leading to fluorescence activation.

**Fig. 4:**
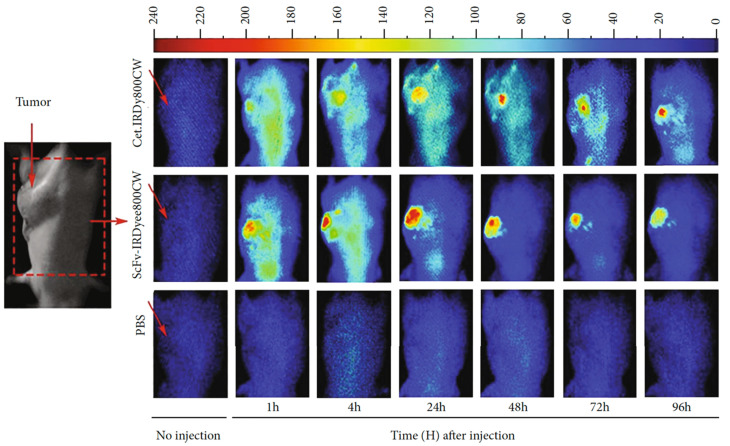
NIR fluorescence images of cet.Hum.scFv-IRDye800CW and cetuximab-IRDye800CW conjugates in mice bearing A-431 tumor xenografts. Images were obtained at no injection and at 1, 4, 24, 48, 72, and 96 hours post-injection of 100 µL for the cet.Hum.scFv-IRDye800CW and cetuximab-IRDye800CW (75 µg). Scale bar changes are shown on the top. The degree of labeling or D/P ratio of cet.Hum.scFv-IRDye800CW and cetuximab-IRDye800CW was 1.983 and 2.128, respectively. Reproduced from Amini A., Safdari Y., Tash Shamsabadi F., Near-Infrared Fluorescence Imaging of EGFR-Overexpressing Tumors in the Mouse Xenograft Model Using scFv-IRDye 800CW and Cetuximab-IRDye 800CW, *Molecular Imaging*, 2022, **2022**, 9589820, under the terms of the Creative Commons Attribution 4.0 International License (CC BY 4.0). https://doi.org/10.1155/2022/9589820.

In 2022, Amini *et al.* introduced cet.Hum.scFv-IRDye800CW, a fluorescent probe designed to improve tumor imaging of EGFR-overexpressing cancers, including head and neck squamous cell carcinoma.^59^ Although full-length antibodies like cetuximab (140–150 kDa) have proven effective in targeting EGFR, their large size can limit solid tumor penetration and prolong blood circulation, leading to higher background fluorescence and thus delayed peak tumor-to-background ratios. To address these issues, the authors created a single-chain variable fragment (scFv) derived from cetuximab, termed cet.Hum.scFv, conjugating it with the near-infrared dye IRDye800CW. Initial *in vitro* assays, including western blot and ELISA, confirmed that both the scFv and full-length cetuximab retain strong, specific binding to EGFR-overexpressing A431 cells but not to EGFR-low U-87 cells. When tested *in vivo* using A431 xenograft mouse models, the scFv-based probe produced a more intense, focused tumor signal than its full-length counterpart [Fig. 3 and 4]. Compared with cetuximab-IRDye800CW, cet.Hum.scFv-IRDye800CW achieved high tumor fluorescence at an earlier time point, with clear differences becoming apparent from 24 h post-injection onward. Moreover, it consistently yielded higher tumor-to-background ratios at every assessed time point (1, 4, 24, 48, 72, and 96 hours). Its TBR peaked at 7.1, significantly outperforming cetuximab-IRDye800CW, which achieved a lower TBR of 5.8. This enhancement was attributed to cet.Hum.scFv's faster clearance from non-target tissues. In contrast, neither probe effectively labeled low-EGFR tumors in U-87 mouse models, underscoring the reliance on strong EGFR expression for robust fluorescence contrast.

The scFv's smaller size explains these performance gains: faster clearance from the bloodstream reduces non-specific background accumulation and accelerates the delivery of an intact probe to tumor tissue. While cetuximab continues to demonstrate robust tumor targeting, its larger molecular weight delays peak signal intensity and can lead to higher systemic background. Because near-infrared light penetrates tissue only to a limited depth, superficial tumors—including many head and neck squamous cell carcinomas—represent ideal indications for these scFv-based probes.

In 2022, Ling *et al.* introduced SQ890 NPs-Pep, a dual-function fluorescent nanoprobe and photothermal conversion agent (PTA) specifically designed for imaging and treating oral squamous cell carcinoma (OSCC).^30^ At its core is SQ890, a near-infrared-II (NIR-II) dye that emits fluorescence in the 1000–1700 nm range, thereby achieving deeper tissue penetration and enhanced contrast for imaging. SQ890 also demonstrates strong absorption around 890 nm, translating into high photothermal conversion efficiency and making it an ideal candidate for combined fluorescence-photoacoustic imaging and photothermal therapy (PTT). To construct the nanoplatform, the SQ890 dye was encapsulated within nanoparticles formed from DSPE-PEG5K-COOH and then conjugated to an EGFR-targeting polypeptide (GE-11). The resulting nanoparticles were spherical, measuring approximately 144–157 nm in diameter. This modification imparts both passive and active tumor targeting, leveraging the enhanced permeability and retention (EPR) effect alongside specific EGFR binding. *In vivo* NIR-II fluorescence imaging performance was assessed in mice bearing CAL 27 oral cancer xenografts. The imaging results showed that SQ890 NPs-Pep produced distinct tumor fluorescent signals with good contrast. BR peaked within hours post-injection, ensuring clear tumor visualization for both imaging and therapeutic purposes. Both passive and active targeting methods resulted in observable tumor fluorescence, but the actively targeted group demonstrated the strongest fluorescent signal as early as 4 hours post-injection, compared to 8 hours for the other groups. Under fluorescence-guided navigation, tumor resection was successfully performed. After surgery, negligible fluorescence signals were detected in the operated region, indicating complete removal of the tumor tissue. *Ex vivo* imaging of excised tumors further confirmed that the targeting formulation delivered much brighter FL signals in the tumor area, validating the specificity of SQ890 NPs-Pep for EGFR-overexpressing cancer cells. The photothermal effect of the NPs was evaluated under NIR light irradiation, leading to a temperature increase sufficient for tumor ablation. PTT experiments showed effective tumor growth suppression with minimal damage to surrounding healthy tissue. Biosafety assessments indicated no significant toxicity in major organs, further supporting the clinical potential of SQ890 NPs. The combination of targeted imaging and effective photothermal treatment highlights the versatility and utility of SQ890 NPs in precision oncology.

In 2021, Wang *et al.* evaluated the EGFR-targeted fluorescent probe ABY-029 for FGS of HNSCC using a paired-agent imaging (PAI) approach.^53^ ABY-029 consists of an anti-EGFR affibody molecule conjugated to IRDye800CW and was developed to provide rapid tumor visualization while overcoming some of the limitations associated with larger antibody-based probes. Due to its small molecular size, ABY-029 exhibits rapid tumor penetration and faster clearance from circulation, enabling imaging within hours rather than days after administration. The study aimed to improve tumor delineation by combining ABY-029 with the untargeted fluorescent tracer IRDye 700DX, thereby generating quantitative binding-potential maps that more accurately reflected EGFR expression while minimizing the effects of nonspecific uptake. The authors employed orthotopic tongue xenograft models using FaDu and Detroit 562 human squamous cell carcinoma cell lines, as well as A431 epidermoid carcinoma cells exhibiting different levels of EGFR expression. Fluorescence imaging was performed 3 h after intravenous administration, with additional experiments evaluating imaging performance between 15 min and 5 h post-injection. Compared with conventional single-agent imaging using ABY-029 alone, the paired-agent approach significantly improved tumor discrimination, particularly in tumors with low or heterogeneous EGFR expression. Diagnostic accuracy increased from 85.4% to 90.8% in FaDu tumors and from 82.2% to 90.8% when all tumor models were analyzed together. Furthermore, the paired-agent method achieved higher receiver operating characteristic curve area under the curve values and demonstrated a stronger correlation with EGFR immunohistochemistry than conventional fluorescence imaging. These findings suggest that ABY-029 is a promising EGFR-targeted probe for FGS of HNSCC and that quantitative paired-agent imaging may further improve the accuracy of intraoperative tumor delineation.

#### 5.2.2. PARP1 and PARP2

Poly(ADP-ribose) polymerases 1 and 2 (PARP1 and PARP2) are enzymes involved in DNA repair processes. Their role in responding to DNA damage makes them critical targets for cancer therapy. Targeting PARP enzymes can lead to synthetic lethality in these cancers. Additionally, these receptors can be exploited in FGS due to their overexpression in certain cancer cells.^73–77^

In 2020, Franca *et al.* introduced PARPi-FL, a small-molecule fluorescent probe designed to specifically target PARP1 and PARP2.^61^ Leveraging the fact that PARP levels are elevated in various cancers, including oral squamous cell carcinoma (OSCC). The synthesis of PARPi-FL involved conjugation of a BODIPY-FL NHS-ester fluorophore to a known PARP inhibitor scaffold, allowing the probe to retain its enzyme-binding characteristics while gaining fluorescent properties. The probe's structure allows it to permeate cell and nuclear membranes, binding to PARP1/2 and generating fluorescence signal in tumors. PARP1, an enzyme overexpressed in OSCC, was firstly confirmed as a viable biomarker through immunohistochemical analysis. PARPi-FL selectively accumulated in tumors and metastatic lymph nodes, while showing minimal uptake in normal tissues. When administered intravenously to mice bearing oral cancers, PARPi-FL accumulated predominantly in tumor tissue, showing little to no fluorescence in surrounding healthy muscle. Quantitatively, the tumor-to-background ratio reached 7.6 overall (10.3 for FaDu and 5.1 for CAL27 xenografts), demonstrating the probe's high specificity and contrast. Its high specificity was further validated by surgical models in which fluorescent imaging identified compromised margins containing residual tumor cells, while disease-free margins displayed negligible fluorescence. A handheld confocal microscope confirmed that PARPi-FL's fluorescence correlated with areas of high PARP1 expression, consistently sparing normal tissues. Immunohistochemistry with an anti-PARP1 antibody supported these findings. PARP1 levels were significantly higher in tumor regions, correlating with areas where PARPi-FL fluorescence was prominent. Conversely, normal muscle tissue exhibited lower PARP1 expression and negligible PARPi-FL fluorescence. This integrated histological and imaging approach offered robust evidence that PARPi-FL can serve as an accurate molecular marker of tumor boundaries and a useful tool for image-guided surgical procedures. Importantly, PARPi-FL has subsequently progressed into clinical evaluation, with a Phase I study demonstrating the safety and feasibility of topical administration in patients with OSCC, while providing sufficient contrast to distinguish malignant lesions from surrounding healthy mucosa. These findings support the translational potential of PARPi-FL and establish it as one of the clinically investigated targeted fluorescent probes for oral cancer imaging.^54^

#### 5.2.3. Fibroblast activation protein

Fibroblast Activation Protein (FAP) is a cell surface serine protease that is highly expressed on cancer-associated fibroblasts (CAFs) in the tumor microenvironment but is rarely found in normal tissues. This selective expression makes FAP a promising target for cancer therapies and imaging techniques, including FGS.^78–83^

In 2023, Li *et al.* introduced [68Ga]Ga-FAP-2286-ICG, a dual-modality probe engineered for both PET and near-infrared (NIR) fluorescence imaging of HNSCC.^62^ The construct comprises FAP-2286—a cyclic peptide that targets the FAP, which is overexpressed in CAFs within the tumor microenvironment of HNCs, conjugated to the FDA-approved fluorophore ICG and chelated *via* DOTA to incorporate the radionuclide 68Ga. This combination enables both PET and NIR imaging capabilities in a single agent. Optical spectra measurements revealed distinct absorption peaks for [68Ga]Ga-FAP-2286-ICG at around 786–779 nm and strong NIR-I emission peaking near 819 nm. The probe's safety profile was confirmed by toxicity assessments in BALB/c mice, showing no signs of acute toxicity over a three-week period. The probe demonstrated high stability, strong FAP specificity, and significant tumor uptake *in vitro*, as confirmed in HNSCC cell models. *In vivo* imaging studies using FaDu, CAL27, and CNE2 tumor xenografts showed that one-hour post-injection, PET/CT scans detected robust and heterogeneous tumor uptake, yielding tumor-to-muscle (T/M) ratios from 2.78 to 6.20. After 72 hours, NIR fluorescence imaging also demonstrated pronounced tumor localization, with tumor-to-background ratios reaching 5.95 ± 2.92 in FaDu and comparable values in CNE2 and CAL27 models. Notably, both PET and fluorescence modalities revealed consistent intratumoral heterogeneity, underscoring the synergy of this dual-imaging system. Clinically, this approach enables preoperative planning using PET/CT to map lesion extent and intraoperative guidance *via* NIR fluorescence for real-time tumor resection. The durable fluorescence signal, maintained over 72 hours, provides scheduling flexibility and potentially enhances surgical precision.

#### 5.2.4. GLUT1

The Glucose Transporter 1 (GLUT1) membrane transporter is a protein that facilitates the transport of glucose across cell membranes. It is overexpressed in many cancer cells due to their increased metabolic demands, making it a potential target for cancer diagnostics and therapies, including FGS.^84–88^

In 2022, Tian *et al.* introduced WZB117-IR820, a fluorescent probe designed to leverage the high expression of the GLUT1 transporter in HNSCC, particularly in OSCC.^60^ The design fused WZB117, a GLUT1 inhibitor, with the near-infrared dye IR820, enabling selective tumor binding and real-time fluorescence imaging. Initial analyses confirmed elevated GLUT1 levels in OSCC cells through western blot and immunohistochemistry of orthotopic OSCC mouse models, with minimal expression in surrounding healthy tissues, underlining GLUT1's potential as a surgical biomarker. Subsequent *in vitro* assays in OSCC cell lines (HSC3, CAL27-fLUC) demonstrated significantly higher fluorescence signals than in healthy oral cells. These cancer cells displayed intense membrane-localized fluorescence within two hours of probe incubation, reflecting strong affinity for GLUT1-overexpressing cells. In an orthotopic OSCC mouse model, *in vivo* imaging showed that fluorescence signals emerged by 30 minutes post-injection and peaked around four hours, delivering an optimal tumor-to-background ratio for surgical guidance. A blocking dose of free WZB117 substantially reduced tumor fluorescence, confirming that the observed signal was GLUT1-dependent. FGS performed at the four-hour mark successfully delineated tumor margins on the tongue, enabling precise resection with no residual disease as verified by postoperative imaging and histopathology. Given that near-infrared light is best suited for superficial lesions such as those in the oral cavity, these findings position WZB117-IR820 as an effective, minimally invasive tool to enhance surgical precision. In sum, by demonstrating robust GLUT1-specific uptake, rapid tumor localization, and clear surgical margins in an animal model, Tian *et al.* highlight GLUT1's suitability as a molecular target and present WZB117-IR820 as a promising probe for improving FGS in oral cancers.

#### 5.2.5. Hsp70

The Heat Shock Protein 70 (Hsp70) is a highly conserved molecular chaperone that assists in protein folding, stabilization, and transport under both normal and stress conditions. In cancer, Hsp70 is frequently overexpressed, promoting tumor cell survival by inhibiting apoptosis and supporting the stability of oncoproteins. It is also found on the surface of tumor cells, where it can be selectively targeted for diagnostic and therapeutic applications, including imaging and immunotherapy.^89–91^ Due to its tumor-specific membrane localization, Hsp70 is an emerging biomarker and target in oncologic imaging strategies.^92,93^

**Fig. 5:**
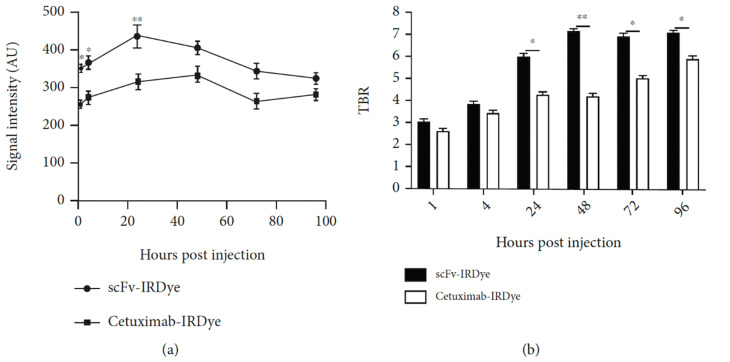
NIR fluorescence analysis of IRDye800CW-conjugated molecules after applying on A-431 tumor-bearing mice. (a) Intensity of fluorescent signals emitting from tumor tissues. (b) Tumor-to-background ratio analysis. Error bars represent mean ± standard deviation. The asterisks indicate significant differences between groups (*p < 0.05, **p < 0.01). Reproduced from Amini A., Safdari Y., Tash Shamsabadi F., Near-Infrared Fluorescence Imaging of EGFR-Overexpressing Tumors in the Mouse Xenograft Model Using scFv-IRDye 800CW and Cetuximab-IRDye 800CW, *Molecular Imaging*, 2022, **2022**, 9589820, under the terms of the Creative Commons Attribution 4.0 International License (CC BY 4.0). https://doi.org/10.1155/2022/9589820.

**Fig. 6:**
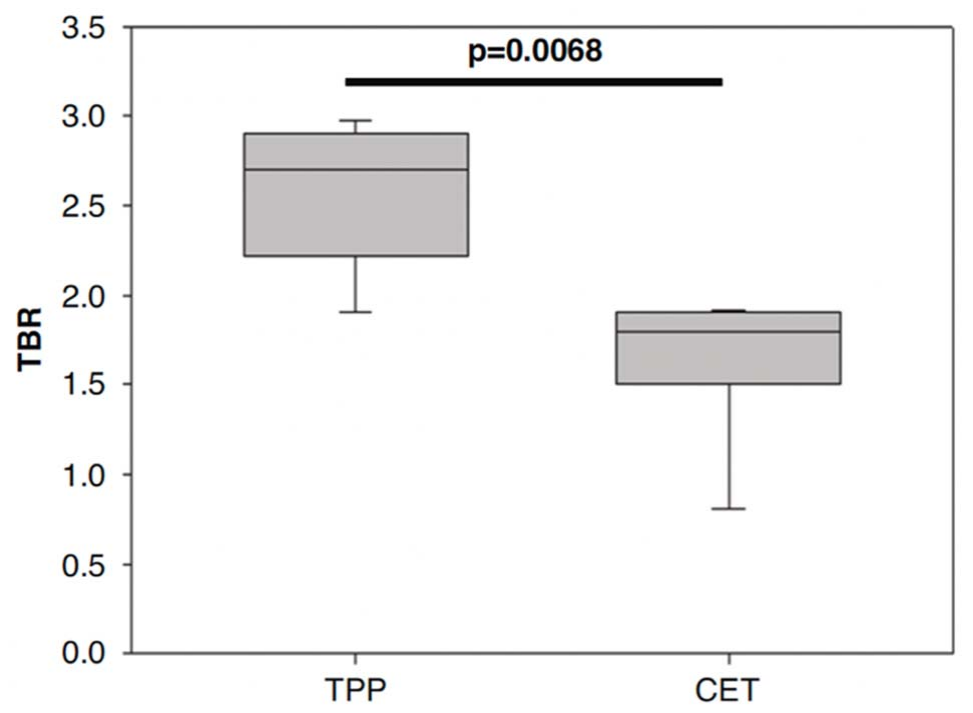
Comparison of the tumor-to-background ratios (TBR) of TPP-IRDye800 *versus* Cetuximab-IRDye680. Significantly higher TBRs (*p* = 0.0068) were detected for TPP-IRDye800 compared to Cetuximab-IRDye680. Data of 7 different HNSCC patients are shown as box plots. Reproduced from Holzmann K. L. K., Wolf J. L., Stangl S., Lennartz P., Kasajima A., *et al.*, “Improved *ex vivo* fluorescence imaging of human head and neck cancer using the peptide tracer TPP-IRDye800 targeting membrane-bound Hsp70 on tumor cells”, *British Journal of Cancer*, 2024, **131**, 1814–1824, under the terms of the Creative Commons Attribution 4.0 International License (CC BY 4.0). https://doi.org/10.1038/s41416-024-02872-8.

In 2024, Holzmann *et al.* introduced a membrane Hsp70-targeting fluorescence probe, TPP-IRDye800, designed for real-time intraoperative imaging of HNSCC and lymph node (LN) metastases.^94^ This tracer is based on a 14-mer tumor cell-penetrating peptide (TPP, TKDNNLLGRFELSG) that mimics the Hsp70 oligomerization domain and specifically binds to membrane-bound Hsp70 (mHsp70), a tumor-selective marker absent in normal tissues. The authors compared the performance of TPP-IRDye800 to the FDA-approved EGFR-targeting Cetuximab-IRDye680 [Fig. 5 and 6].

Flow cytometry and fluorescence microscopy using HNSCC cell lines (CAL-27 and UD-SCC-5) and patient-derived primary tumor samples confirmed mHsp70 expression and efficient TPP uptake. For *ex vivo* imaging, freshly resected tumor and LN specimens were sequentially sprayed with both tracers. TPP-IRDye800 showed significantly higher tumor-to-background ratios (TBR = 2.56 ± 0.39) compared to Cetuximab (TBR = 1.61 ± 0.39, *p* = 0.0068), with sharper tumor margin delineation.

Histopathology confirmed tracer specificity. Importantly, the tracer enabled detection of carcinoma *in situ* and LN metastases, while negative nodes remained unstained. Imaging results were validated on clinical-grade systems, and in a murine model, topical application of TPP-IRDye800 selectively stained tumors but not normal organs. The authors highlighted key advantages of topical application, rapid imaging, minimal systemic exposure, and suitability for intraoperative use, although noted limitations include superficial penetration, signal artifacts at electrosurgical borders, and the need for repeated spraying. As a next step, intravenous application in humans is planned to overcome these challenges and further improve clinical translation.

### 5.3. Activatable probes

Secondly, we will consider some of the activatable probes that have been investigated in this context.

In 2023, Pola *et al.* developed advanced stimuli-responsive polymer nanoprobes designed for FGS of head and neck tumors.^50^ These nanoprobes are based on p(HPMA) copolymers. By integrating fluorescent dyes (Cy7) with specific spacers, the nanoprobes are engineered to activate fluorescence in response to the unique conditions of the tumor microenvironment, such as acidity or the presence of specific enzymes, thereby increase tumor-to-background ratio.

**Fig. 7:**
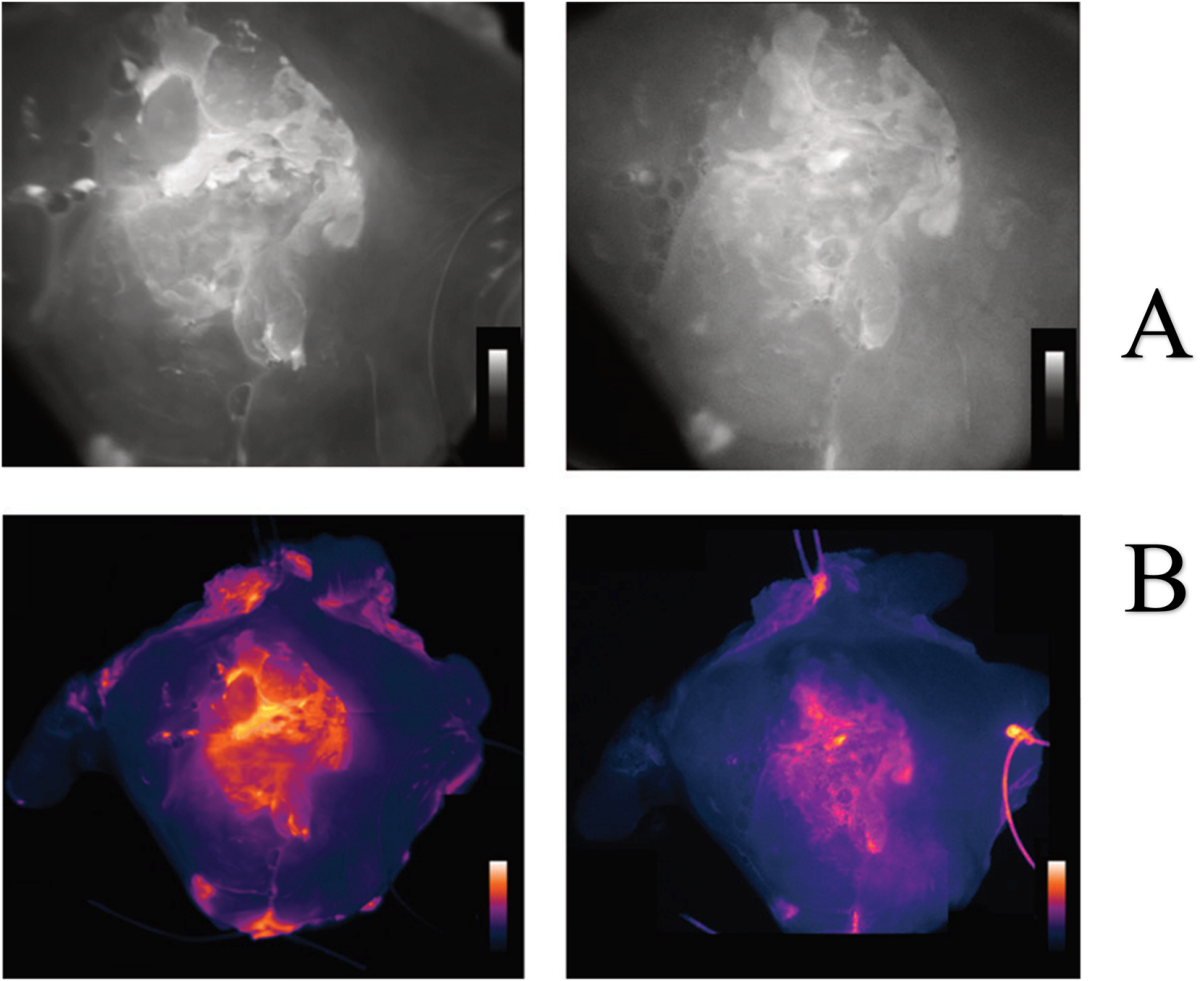
Comparison of the tumor-to-background ratios (TBR) of TPP-IRDye800 *versus* Cetuximab-IRDye680. The fluorescence images (A) show a less diffuse signal enhancement within the tumor and a clearer demarcation of the tumor to the surrounding healthy tissue for TPP-IRDye800 (left) compared to Cetuximab-IRDye680 (right). The pseudo-color overlay (B) reveals a stronger signal accumulation (yellow color code) in the tumor area for TPP-IRDye800 (left) compared to Cetuximab-IRDye680. Signal intensity scales are displayed in each image. Reproduced from Holzmann K. L. K., Wolf J. L., Stangl S., Lennartz P., Kasajima A., *et al.*, “Improved *ex vivo* fluorescence imaging of human head and neck cancer using the peptide tracer TPP-IRDye800 targeting membrane-bound Hsp70 on tumor cells”, *British Journal of Cancer*, 2024, **131**, 1814–1824, under the terms of the Creative Commons Attribution 4.0 International License (CC BY 4.0). https://doi.org/10.1038/s41416-024-02872-8.

**Fig. 8:**
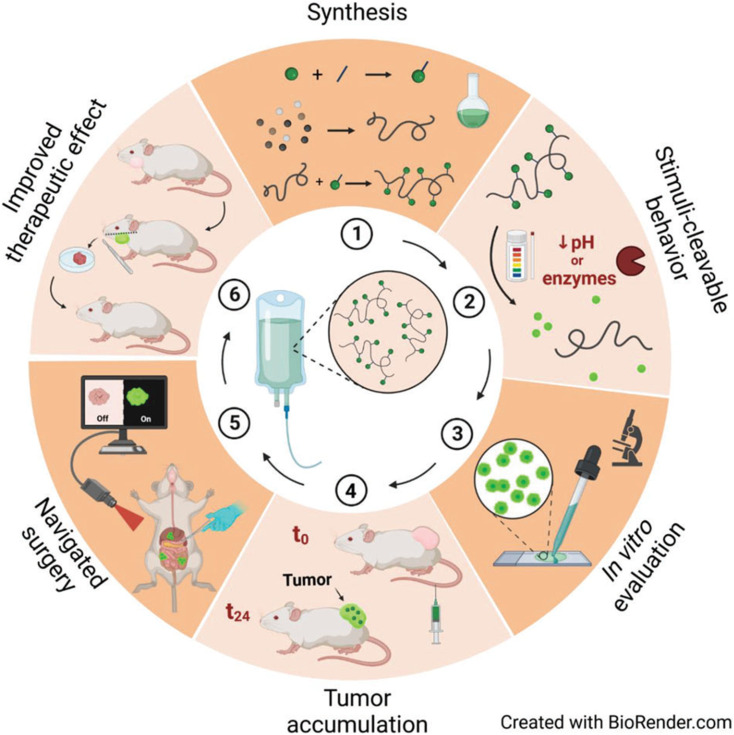
“Life-cycle” scheme of the polymer-probe for fluorescently guided surgery. Reproduced from Pola R., Grosmanová E., Pechar M., Horák D., Krunclová T., Pankrác J., Henry M., Kaňa M., Bouček J., Šefc L., Coll J.-L., Etrych T., “Stimuli-Responsive Polymer Nanoprobes Intended for Fluorescence-Guided Surgery of Malignant Head-and-Neck Tumors and Metastases”, *Advanced Healthcare Materials*, 2023, **12**(28), e2301183, under the terms of the Creative Commons Attribution License (CC BY). https://doi.org/10.1002/adhm.202301183.

Three spacer types were used: pH-sensitive hydrazone bonds, enzymatically degradable bonds sensitive to lysosomal enzymes, and a non-degradable control spacer. The nanoprobes were synthesized with high purity, maintaining a hydrodynamic diameter of 6–10 nm, which is ideal for prolonged circulation and eventual renal excretion. Extensive testing demonstrated the efficacy of the probes in both *in vitro* and *in vivo* models [Fig. 7]. *In vitro* studies with FaDu confirmed that the nanoprobes were efficiently internalized and showed significant fluorescence activation within 24 hours under tumor-like conditions, such as slightly acidic pH or the presence of lysosomal enzymes inside the cells. Among the tested probes, those with enzymatically cleavable linkers exhibited the highest fluorescence intensity. *In vivo* biodistribution studies in mice bearing FaDu tumors revealed significant tumor accumulation and a high TBR, particularly for probes with pH-sensitive and enzymatically cleavable spacers. This enhanced fluorescence contrast ensured accurate identification of tumor margins. The nanoprobes were further evaluated in fluorescence-guided surgical applications. In mouse models of intraperitoneal metastases and orthotopic head and neck tumors, the probes enabled precise tumor detection and resection. Notably, the pH-sensitive probe P-H-OPB-Cy7 outperformed non-degradable controls, reducing tumor burden by 97.2% and achieving a high positive predictive value for tumor localization. The optical guidance provided by these probes allowed surgeons to identify and remove residual tumor tissue that would otherwise be missed under normal lighting conditions (Fig. 8).

In 2022, Chen *et al.* developed and synthetized bioinspired styrene oxazolone dyes (SODs), as promising fluorescent probes for biomedical imaging, particularly in intraoperative imaging of head and neck carcinomas.^37^ These dyes exhibit long Stokes shifts (>130 nm) and NIR emissions (>650 nm), with absorption maxima around 485 nm, which enhance tissue penetration and reduce autofluorescence. Among the SODs, SOD9 and its derivative SOD9-TPP stand out due to their superior optical and biological properties. SOD9, inspired by fluorescent protein chromophores, was synthesized using a straightforward method that ensures high fluorescence intensity and Stokes shifts. It features rapid renal clearance, high quantum yield and also the ability to cross the blood–brain barrier. *In vitro* tests demonstrated strong cellular uptake with negligible toxicity, while *in vivo* studies revealed rapid excretion *via* the renal pathway and significant fluorescence in the brain and bladder within minutes post-injection. The biodistribution of SOD9 indicated primary localization in the brain and gallbladder, with TBR of approximately 3 observed in HNSCC models. To enhance tutor specificity, SOD9 was conjugated with triphenylphosphonium (TPP), creating SOD9-TPP, a mitochondria-targeted probe. This modification amplified tumor-targeting efficiency and altered the excretion pathway to liver clearance. *In vitro* studies showed selective accumulation of SOD9-TPP in tumor cells compared to normal cells, confirming its tumor-specific targeting capability. *In vivo* imaging of HNSCC tumors demonstrated a TBR of up to 3 within 60 minutes post-injection, supporting its potential for precise tumor visualization. Additionally, SOD9-TPP enabled fluorescence-guided tumor resection, simplifying surgical procedures and real-time pathological assessments through confocal microscopy. This approach improved the accuracy of tumor margin identification and reduced surgical trauma.

In 2020, Zou *et al.* introduced Cou-Br, a two-photon fluorescent probe tailored to detect and visualize glutathione (GSH) in living systems, with a particular focus on laryngeal cancer.^57^ GSH is significantly overexpressed in laryngeal cancer compared to normal tissues, making it a potential biomarker for tumor identification and surgical guidance. By exploiting the minimal photodamage and deep tissue penetration of two-photon imaging, Cou-Br can accurately highlight tumor regions during intraoperative procedures. Structurally, the probe comprises a coumarin fluorophore and a brominated recognition unit. In the absence of GSH, the bromine atom quenches fluorescence *via* the heavy-atom effect; however, when GSH is present, bromine is replaced by the thiol group, resulting in a strong fluorescence “turn-on” signal. Importantly, the probe displays only minimal response to other biothiols such as cysteine and homocysteine, thus ensuring selective detection of GSH. The probe's absorption maximum is around 462 nm, and it can be excited at 900 nm under two-photon excitation, facilitating deeper tissue imaging than one-photon probes. *In vitro* studies confirmed Cou-Br's low toxicity and high biocompatibility, effectively imaging GSH in various tumor cell lines, including FaDu cells. The probe's fluorescence increased significantly in the presence of GSH and was reduced when GSH levels were depleted or oxidized. This dynamic response allowed real-time visualization of intracellular GSH fluctuations under oxidative stress. *In vivo* imaging in both subcutaneous and orthotopic laryngeal tumor-bearing mice demonstrated that Cou-Br preferentially accumulated in tumor tissues over time, producing notably stronger fluorescence signals in the tumor regions than in surrounding normal tissues, creating high TBR. This distinction enabled clear demarcation of tumor boundaries. Subsequent histological analyses (H&E staining) confirmed that areas of high Cou-Br fluorescence correlated with the presence of cancer cells, whereas areas with weak fluorescence did not contain tumor cells. To evaluate clinical relevance, human laryngeal carcinoma tissues of different pathological types were incubated with Cou-Br. In all cases, tumor regions exhibited substantially stronger fluorescence signals than adjacent healthy tissues, highlighting Cou-Br's capacity to discriminate malignant lesions.

Activatable probes have demonstrated promising preclinical performance and the potential to improve tumor-to-background ratios compared with conventional always-on probes. However, their broader translation into clinical practice may be challenged by the increased complexity of probe design, the need to ensure reliable activation within heterogeneous tumor environments, and the additional requirements associated with manufacturing, safety evaluation, and regulatory approval. Nevertheless, activatable probes remain a highly promising direction for future FGS due to their potential to further improve tumor specificity and intraoperative contrast.

## 6. Overview of imaging probes

The always-on probes discussed in this review are summarized in [Table 2], and the activatable fluorescent probes are presented in [Table 3].

**Table 2:** Always-on fluorescent probes used in fluorescence-guided surgery for head and neck carcinomas

Probe	Fluorophore	Target	TBR	Model	Imaging time	Stage
Panitumumab-IRDye800CW	IRDye 800CW	EGFR	Not reported	FaDu xenograft mouse model	48 h	Clinical
P-GE11-Cy7	Cyanine 7	EGFR	Not reported	FaDu xenograft mouse model	24 h	Preclinical
cet.Hum.scFv-IRDye800CW	IRDye 800CW	EGFR	7.1	A-431 xenograft mouse model	24 h	Preclinical
WZB117-IR820	IR-820	GLUT1	∼2.1 (not specified)	FaDu xenograft mouse model	4 h	Preclinical
SQ890 NPs-Pep (GE11)	SQ890	EGFR	∼3.5 (not specified)	CAL-27 xenograft mouse model	4 h	Preclinical
PARPi-FL	BODIPY-FL	PARP1/2	10.3 (FaDu), 5.1 (Cal-27)	FaDu and CAL27 xenograft mouse models	90 min	Phase 1 clinical
[68Ga]Ga-FAP-2286-ICG	Indocyanine green	FAP	5.95 ± 2.92 (FaDu), 2.77 ± 1.70 (CAL-27), 5.8 ± 1.15 (CNE2)	FaDu, CAL-27, and CNE2 xenograft mouse models	72 h	Preclinical
TPP-IRDye800	IRDye 800CW	Hsp70	2.56 ± 0.39	Human HNSCC surgical specimens	5 min topical incubation (*ex vivo*)	Clinical *ex vivo* study
ABY-029	IRDye 800CW	EGFR	Not reported	FaDu and Detroit 562 xenograft mouse models	3 h	Phase 0 clinical evaluation

**Table 3:** Activatable fluorescent probes used in fluorescence-guided surgery for head and neck carcinomas

Probe	Fluorophore	Target	TBR	Model	Imaging time	Stage
Cou-Br	Coumarin derivative	GSH	Not reported	FaDu xenograft mouse model and human HNSCC surgical specimens	60 min, 30 min topical incubation (*ex vivo*)	Clinical *ex vivo* study
SOD9-TPP	SOD9	Mitochondrial activity	∼3	SCC090 xenograft mouse model	60 min	Preclinical
P-H-OPB-Cy7	Cyanine 7	Tumor microenvironment	∼2.5 (not specified)	FaDu xenograft mouse model	48 h	Preclinical

## 7. Future prospects and research directions

The future of FGS in HNSCC is expected to be shaped by advances in probe design, imaging technology, and clinical application. One of the most promising directions is the development of activatable probes, which remain quenched until exposed to tumor-specific conditions such as enzymatic activity or acidic pH. These probes have attracted considerable interest because their selective activation may reduce background fluorescence and further improve tumor-to-background contrast compared with conventional always-on probes. By selectively switching on within the tumor microenvironment, these probes could provide higher contrast and more accurate margin delineation than the “always-on” agents currently available.^38,45^ Although activatable probes offer the potential for improved tumor specificity and higher tumor-to-background contrast, their clinical translation in HNSCC will require further validation, standardized imaging protocols, and demonstration of clinical benefit in prospective studies before routine implementation can be achieved.^95–98^ Another anticipated advance is the integration of fluorescence with multimodal imaging platforms, combining real-time visualization with preoperative functional and anatomical data. While already being explored, future hybrid probes that link fluorescence with PET or photoacoustic imaging may allow seamless transition from staging to surgery, providing a comprehensive roadmap for tumor resection.^99^ However, the successful clinical translation of novel fluorescent probes will require not only favorable imaging performance but also demonstration of safety and reproducible manufacturing while maintaining clinical feasibility.^95,97^ Future probe development is also expected to expand beyond EGFR toward additional molecular targets. FAP-targeted dual-modality probes have recently demonstrated promising results in HNSCC-related models, while PARP1-targeted fluorescence imaging has shown clinical feasibility for oral cancer detection.^54^ Other emerging targets, including GLUT1 and membrane-bound Hsp70, have also demonstrated encouraging results in preclinical and early translational studies.^60,94^ Collectively, these developments suggest that future advances in FGS will depend on both the identification of highly specific molecular targets and the successful translation of these imaging agents into routine clinical practice. Looking ahead, FGS is also expected to broaden its scope beyond primary tumor resections. Potential applications include guiding surgery in recurrent disease, detecting submucosal or perineural tumor spread, and introducing theranostic probes that combine tumor visualization with light-activated therapeutic effects. Such approaches could make surgery both more precise and more effective, uniting diagnosis and treatment in a single intervention.^56,60^ Importantly, the current translational status of fluorescent probes in HNSCC remains heterogeneous. While EGFR-targeted probes such as panitumumab-IRDye800CW and cetuximab-IRDye800CW have already progressed to clinical evaluation in patients with HNSCC,^100–102^ many newer probe classes, including activatable, metabolic, and stromal-targeted agents, remain at the preclinical or early translational stage. Similarly, PARP1-targeted imaging has demonstrated encouraging translational progress, with PARPi-FL showing clinical feasibility for oral cancer detection.^54^ Despite these advances, broader clinical implementation will require further validation in prospective clinical studies, standardization of imaging protocols, and demonstration of clear clinical benefit over existing surgical approaches.^95,97^ In summary, continued advances in probe design, target selection, and multimodal imaging are expected to further improve the precision of FGS and facilitate the translation of molecularly targeted imaging into routine clinical practice.

## 8. Conclusions

Fluorescence-guided surgery represents a promising strategy for improving the surgical management of HNSCC by enhancing intraoperative tumor visualization and facilitating more precise margin assessment. Recent advances in fluorescent probe development have driven the field from non-specific fluorophores toward both molecularly targeted and metabolism-based imaging agents with improved tumor specificity and imaging performance. The emergence of targeted, activatable, and multimodal probes has substantially expanded the capabilities of FGS and improved the ability to distinguish malignant tissue from surrounding healthy structures. Overall, EGFR-targeted probes currently represent the most clinically advanced class of fluorescent imaging agents for HNSCC, with panitumumab-IRDye800CW, cetuximab-IRDye800CW, and ABY-029 already evaluated in clinical studies. Among clinically investigated alternatives, 5-ALA has also demonstrated feasibility for intraoperative visualization of HNSCC, supporting the potential of non-receptor-targeted imaging strategies. Among emerging approaches, PARP-targeted probes have demonstrated particularly strong translational potential, achieving tumor-to-background ratios of up to 10.3 in FaDu xenografts and 7.6 overall while also progressing to Phase I clinical evaluation. FAP-targeted probes have shown promising imaging performance in preclinical studies and offer a unique strategy for targeting the tumor microenvironment rather than malignant cells directly. Activatable probes may provide further improvements in tumor specificity and tumor-to-background contrast through selective activation within malignant tissue, potentially overcoming some of the limitations associated with conventional always-on probes. In parallel, theranostic approaches integrating fluorescence imaging with photodynamic or photothermal therapy may expand the role of FGS beyond tumor visualization alone, enabling simultaneous tumor detection and treatment. Despite substantial progress, challenges related to clinical validation, standardization of imaging protocols, manufacturing complexity, and routine clinical implementation remain. Continued advances in probe design, target selection, imaging technology, and prospective clinical evaluation will be required to fully realize the potential of FGS. Collectively, the evidence reviewed here suggests that EGFR-targeted probes currently offer the most mature pathway toward clinical implementation, whereas PARP-targeted, FAP-targeted, activatable, and theranostic imaging strategies represent particularly promising directions for future development. The combination of clinically validated targeted probes with next-generation activatable and multimodal imaging agents is likely to define the future of FGS in HNSCC.

## Conflicts of interest

The authors declare no conflict of interest.

## Data Availability

No new data were generated or analysed in this study. All information is derived from previously published studies cited in the article.
